# Sample size issues in multilevel logistic regression models

**DOI:** 10.1371/journal.pone.0225427

**Published:** 2019-11-22

**Authors:** Amjad Ali, Sabz Ali, Sajjad Ahmad Khan, Dost Muhammad Khan, Kamran Abbas, Alamgir Khalil, Sadaf Manzoor, Umair Khalil

**Affiliations:** 1 Department of Statistics Islamia College, Peshawar, Pakistan; 2 Department of Statistics, Abdul Wali Khan University Mardan, Pakistan; 3 Department of Statistics, University of Azad Jammu & Kashmir, Muzaffarabad, Pakistan; 4 Department of Statistics, University of Peshawar, Pakistan; Tongii University, CHINA

## Abstract

Educational researchers, psychologists, social, epidemiological and medical scientists are often dealing with multilevel data. Sometimes, the response variable in multilevel data is categorical in nature and needs to be analyzed through Multilevel Logistic Regression Models. The main theme of this paper is to provide guidelines for the analysts to select an appropriate sample size while fitting multilevel logistic regression models for different threshold parameters and different estimation methods. Simulation studies have been performed to obtain optimum sample size for Penalized Quasi-likelihood (PQL) and Maximum Likelihood (ML) Methods of estimation. Our results suggest that Maximum Likelihood Method performs better than Penalized Quasi-likelihood Method and requires relatively small sample under chosen conditions. To achieve sufficient accuracy of fixed and random effects under ML method, we established ‘‘50/50” and ‘‘120/50” rule respectively. On the basis our findings, a ‘‘50/60” and ‘‘120/70” rules under PQL method of estimation have also been recommended.

## Introduction

Individuals, who are drawn from a hospital, school or a classroom, tend to share more homogeneity as compared to those drawn from a population which is very large in size. As such individuals will always enjoy various common properties like family background, morals and values, religion, socio-economic status, demographic, etc., complete independence of observations in such situations is never going to happen [1]. If we have nested data or multilevel data, the assumption of independence will be clearly violated and the application of analysis of variance (ANOVA) and linear regression will be incorrect because these two classical models assume independence, so substitute statistical models (Multilevel Models) needed to examine and analyze such nested data [2]. Most of the time the data is in the form of multilevel data structure like in hospitals and educational institutions, and for this type of data researchers frequently used statistical models called multilevel models, hierarchical models, mixed effects models [3], [4], which are gaining recognition very rapidly. For the last 10 years, these models have become much admired and still are on rise in terms of popularity among researchers in various fields. As one of the prime questions in any field of research is to decide about an appropriate sample size, the decision and issues regarding sample size are not very straight forward in multilevel modeling. Therefore, for a quantitative study, the decision about the optimum sample size can be extremely tricky due to estimation complexity of the models and the size of the sample at each level. The issues of sample size in multilevel models have been discussed by various researchers for continuous response variable. According to [5] for a model having fixed coefficients, the group size of less than 10 is enough. However, for random coefficients a group size of ≥ 10 is needed. [6] Concluded that to get high power and accuracy one should use more level-2 units than level-1 units. Similarly, for level 2 effects and cross-level interactions the power of the test mainly depends on level 2 units. [7] carried out simulation study regarding sample size issues in multilevel models for a continuous response variable and they determined that for fixed effects 10 groups are sufficient, for contextual effects 30 groups are essential and for valid estimation of standard errors 50 groups are required. Similarly, Maas and Hox [8] carried out another simulation study for a continuous response variable by taking three groups (30, 50,100), three group sizes (5, 30, 50) and Intra Class Correlation i.e. ICC (0.1, 0.2, 0.3). They concluded that across all simulated conditions, the estimates were unbiased and reported under estimation of level 2 variance components when number of groups was below 100. It was also concluded that for better estimation at least a sample of size 100 is needed for level 2. However, there is fewer research conducted in the context of binary response variable. Moineddin et al., [9] performed simulation study for the determination of sample size for multilevel binary logistic regression model with single level-1 explanatory variable and single level-2 explanatory variable and by taking three groups conditions(30,50,100), three group sizes (5,30,50) and ICC (0.04,0.17,0.38). They came to the conclusions that when the number group is equal to hundred with a group size of fifty or more, the fixed effect parameters estimates were unbiased. Secondly, when the number group is equal to hundred with a group size of fifty, the variance components were reported to have a bias. The amount of bias was extremely high for the random effects as well as for the fixed effect when the group size was five. The standard errors for the variance components were underestimated and for fixed effect parameters they were unbiased. Paccagnella [10] used a multilevel binary logistic random intercept model in the simulation study and explored that similar to continuous response variable model, the bias of fixed parameter estimates decreased with increase in number of groups. Acceptable coverage rates were achieved for fixed effects estimates when number of groups was 50. Unlike continuous outcome variable models, a very large number of groups is needed to achieve acceptable coverage rates for the variance components estimates.

Zeng [11] proposed a Bayesian spatial generalized ordered logit model to analyze freeway crash severity. The suggested model was superior as compared to the traditional generalized ordered logit model in terms of statistical significance of the spatial term and better model fit. Similarly, to analyze crash rate by injury severity, three temporal multivariate random parameters Tobit models were developed by Zeng [12]. In all of the temporal models, significant temporal effects are found and the goodness of fit (Bayesian *R*^2^) of the multivariate random parameters, Tobit regression improves considerably due to the inclusion of temporal correlation. The inclusion of spatio-temporal correlation and interaction in a multivariate random-parameters Tobit model and their influence on fitting arial crash rates with different severity outcomes have been investigated by [13] in the Bayesian context. The proposed model performs better in terms of model fit than a multivariate random-parameters Tobit model and a multivariate random parameters spatial Tobit model.

A driving simulator experiment was conducted by [14] to investigate the safety of the truck under crosswind at the bridge-tunnel section. Steering angle and the yawing rate were the indices of the dynamic response under crosswinds. To prevent the possible accident, the authors recommended various safety options. In another study, [15] used Mixed logit models to reveal random effects. This was the first ever investigation of the difference in driver-injury severity between single vehicle (SV) and multi-vehicle accidents (MV). Respective critical risk factors of SV and MV accidents were evaluated and compared. Comprehensive observations, which have not been covered in the existing studies, were made. Additionally, to examine factors affecting injury sustained by two drivers involved in the same rear-end crash between passenger cars, a random parameters bivariate ordered probit model has been developed by Chen [16]. The proposed model outperforms the two separate ordered probit models with fixed parameters.

In multilevel models small group sizes such as 5, 10, and 15 and 20 are usually considered in education, behavioral science, etc. But here, large group number and moderate group sizes have been utilized. As compared to the linear multilevel models, larger group numbers are needed for multilevel logistic regression models. That is why small group number has been ignored in this study. Moreover, Bayesian methods may also be very useful in such situations, and can reduce model misspecification and estimation bias significantly.

So far, very little research has been conducted in the literature regarding sample size determination in the context of multilevel logistic regression models. For example, there is nothing in the contemporary research about PQL method in multilevel logistic regression models. Therefore, the present study attempts to capitalize on a novel state-of-the-art rule that encompasses all the weaknesses of the available methods for both fixed and random effects estimates under ML and PQL methods of estimation. In addition, the present study also provides guidelines about optimum sample size needed for multilevel logistic regression models. Random intercept and random slope model with two level-1 and one level-2 explanatory variables using threshold parameter concept are used. Further, larger random effects are incorporated in the present study which were unnoticed in the previous literature. Moreover, relevant factor and their levels were also ignored in [9] and [10]. A detailed comparison of ML method and PQL method has been made in terms of sample size.

## Materials and methods

Multilevel Logistic Regression Model:

A very popular concept is used in social sciences to develop a dichotomous multilevel logistic model through a latent continuous variable model [17]. A threshold concept is used that the latent continuous variable $Y_{ij}^{*}$ underlies the observed variable *Y*_*ij*_. A simple two level dichotomous model is

$$\begin{array}{l} Y_{ii}^{*}=\beta_{0j}+\beta_{1j}X_{1ij}+\beta_{2}\;X_{2ij}+e_{ij}\;\mathrm{Level}\;1\;\mathrm{model}\\ \;\beta_{0j}=\gamma_{00}+\gamma_{01}\;W_{j}+u_{oj}\;\mathrm{Level}\;2\;\mathrm{model}\\ \;\beta_{1j}=\gamma_{10}+\gamma_{11}\;W_{j}+u_{1j}\\ \;\beta_{2}=\gamma_{20} \end{array}$$ (1)

The combined model was obtained by substituting level 2 model in level 1 model:

$$\begin{array}{l} Y_{ij}^{*}=\left(\gamma_{00}+\gamma_{10}X_{1ij}+\gamma_{20}X_{2ij}+\gamma_{01}\;W_{j}+\gamma_{11}X_{1ij}W_{j}\right)+\left(u_{oj}+u_{1j}X_{ij}+e_{ij}\right)\\ \;\left(\mathrm{Fixed}\;\mathrm{part})\;+(\mathrm{Random}\;\mathrm{part}\right) \end{array}$$ (2)

Where *X*_1*ij*_ and *X*_2*ij*_ are the Level-1 explanatory variables, *W*_*j*_ is the Level-2 explanatory variable, Level 1 coefficients denoted by *β* and *γ*′*s* are the fixed effects. If *e*_*ij*_∼ logistic (0,*π*^2^/3), the model is, then, referred to as multilevel logistic model [18]. Random effects of level 2 assumed to have a multivariate normal distribution

$$\left[\begin{array}{c} u_{oj}\\ u_{1j} \end{array}\right]∼N\left(\left[\begin{array}{c} 0\\ 0 \end{array}\right],\left[\begin{array}{cc} \sigma_{u}^{2} & \sigma_{u1}\\ \sigma_{u1} & \sigma_{1}^{2} \end{array}\right]\right)$$ (3)

It should be noted that the equation for Intra Class Correlation (ICC) is

$$ICC=\sigma_{u}^{2}/{(\sigma }_{u}^{2}+\pi^{2}/3)$$ (4)

According to [19], ICC is an estimate of the total variance explained by the grouping structure.

Now $Y_{ij}^{*}$ can be linked with the observed variable *Y*_*ij*_ through a threshold *γ*, and this threshold is also the intercept of the above model. As we have only two categories, so

$$Y_{ij}=0\;if\;Y_{ij}^{*}\leq \gamma \;\mathrm{and}\;Y_{ij}=1\;if\;Y_{ij}^{*}>\gamma .$$

The other concept or approach towards multilevel logistic regression models is that of Multilevel Generalized Linear Models. Both approaches lead to equivalent models, but certainly different at the conceptual level.

Let *Y*_*ij*_ be a binary response variable representing the occurrence or nonoccurrence of some characteristics having values 0 and 1, corresponding to the individual level unit (*i* = 1,2……*nj*, j = 1,2……*N*) and *i* is nested in *j*. A multilevel dichotomous logistic model with two level 1 explanatory variables and single level 2 explanatory variable can be written as

$$\begin{array}{l} \mathrm{logit}\;\left(P_{ij}\right)=\beta_{0j}+\beta_{1j}X_{1ij}+\beta_{2}X_{2ij}\;\mathrm{Level}\;1\;\mathrm{model}\\ \;\beta_{0j}=\gamma_{00}+\gamma_{01}\;W_{j}+u_{oj}\;\mathrm{Level}\;2\;\mathrm{model}\\ \;\beta_{1j}=\gamma_{10}+\gamma_{11}\;W_{j}+u_{1j}\\ \;\beta_{2}=\gamma_{20} \end{array}$$ (5)

The combined model was obtained by substituting level 2 model in level 1 model:

$$\begin{array}{l} \mathrm{logit}\left(P_{ij}\right)=\left(\gamma_{00}+\gamma_{10}X_{1ij}+\gamma_{20}X_{2ij}+\gamma_{01}\;W_{j}+\gamma_{11}\;X_{1ij}W_{j}\right)+\left(u_{oj}+u_{1j}X_{1ij}\right)\\ \;\left(\mathrm{Fixed}\;\mathrm{part})\;+(\mathrm{Random}\;\mathrm{part}\right) \end{array}$$ (6)

This particular model was utilized in the present study.

It should be noted that the lowest error *e*_*ij*_ is absent in the Eq (1) because it is part of Generalized Linear Model specification [20]. This particular framework of generalized linear model is very popular in biostatistics.

It would be easier to formulate the equation as *P*_*ij*_ = expit (regression equation) where expit is the inverse of the logit function [6]. Then for simulation studies, one would have to specify the mean and variance of all predictor variables, and the values of all regression coefficients. The predictor variables would be randomly generated, and the expit function would turn the continuous prediction into a proportion, which can then be dichotomized according to the chosen threshold. Similarly, one can use the following expression

$$P_{ij}=\exp\;\left(\beta_{0j}+\beta_{1j}X_{1ij}+\beta_{2}X_{2ij}\right)/1+\left\{\exp\;\left(\beta_{0j}+\beta_{1j}X_{1ij}+\beta_{2}X_{2ij}\right)\right\}$$ (7)

## Simulation design

The threshold parameter was set to (*γ*_00_ = -1.22), corresponding to approximately twenty percent prevalence rate of the response variable. Similarly, the other fixed effect parameters (*γ*_10_,*γ*_20_,*γ*_01_,*γ*_11_) were set on the analogy of the studies conducted by [7] and [9]. That is *γ*_10_ = 0.3, *γ*_20_ = 0.3, *γ*_01_ = 0.3, *γ*_11_ = 0.3. The explanatory variables (*X*_1*ij*_,*X*_2*ij*_ *and* W_j_) were all generated from standard normal distribution. $u_{0j}∼N(0,\sigma_{u}^{2})$ and $u_{1j}∼N(0,\sigma_{1}^{2})$, where $\sigma_{u}^{2}$ follows from intra-class correlation specification. The $\sigma_{1}^{2}$ value was set to 1 in all simulation scenarios and for simplicity, the term *σ*_*u*1_ was set to zero.

Four scenario for the number of group’s factor and three each for group size and ICC were used. The number of Groups were taken as (30, 50,100 and 120), Group sizes were (5, 30 and 50) and ICC were set to be (0.1, 0.2 and 0.4). It means, we have 4×3×3 = 36 scenarios and for each one, the number of simulations “R” was set to be 1000.

## Analysis

The accuracy of different fixed effect and random effect parameters estimates were calculated through the relative bias = (estimate-parameter)/parameter. Empirical coverage rates of 95% confidence intervals were used to judge the accuracy of the standard errors of estimated parameters. The 95% confidence intervals coverage rates were computed in each scenario as the proportion of replications in which the true parameter is captured by the 95% confidence interval. Bradley recommended acceptable coverage rates as 92.5% to 97.5% [21]. Empirical powers were computed for the *X*_1*ij*_*X*_2*ij*_, *W*_*j*_, and *X*_1*ij*_×*W*_*j*_. The power was calculated as the number of replications in which *H*_0_ of null effect was correctly rejected at 5 percent level of significance divided by 1000 as 1000 replications was used for each scenario. Moreover, a separate logistic regression was used to judge the influence of various simulation scenarios on estimates empirical coverage rates.

## Results

**Fig 1:**
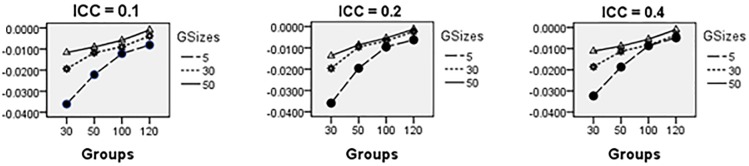
Average relative bias for the estimate of the threshold parameter across all conditions (ML method).

**Fig 2:**
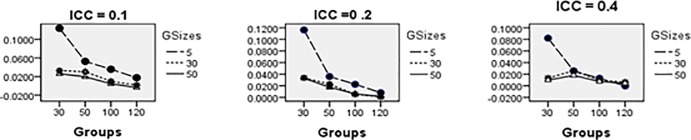
Average relative bias for the estimate of level 1 variable *X*_1*ij*_ coefficient across all conditions (ML method).

**Fig 3:**
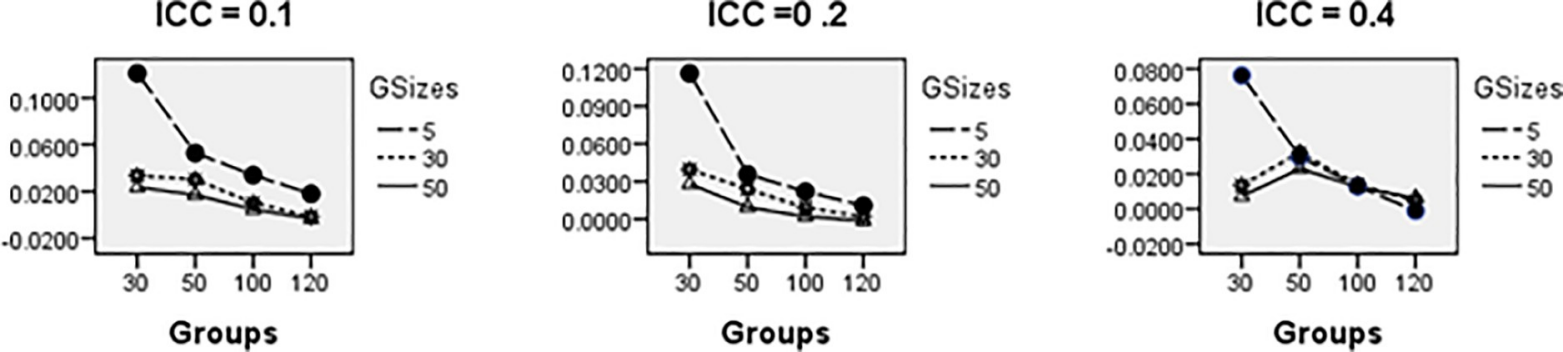
Average relative bias for the estimate of level 1 variable *X*_2*ij*_ coefficient across all conditions (ML method).

**Fig 4:**
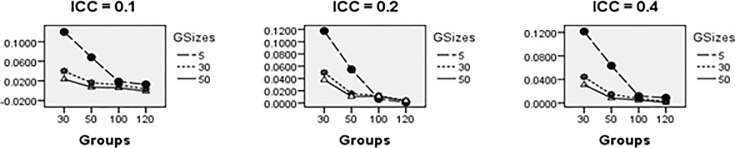
Average relative bias for the estimate of level 2 variable *W*_*j*_ coefficient across all conditions (ML method).

**Fig 5:**
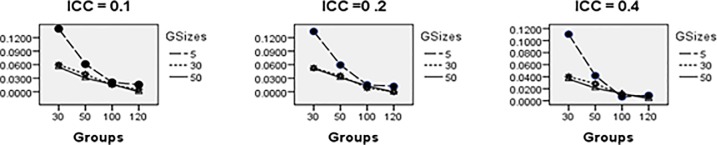
Average relative bias for the estimate of cross-level interaction *X*_1*ij*_*W*_*j*_ coefficient across all conditions (ML method).

**Fig 6:**
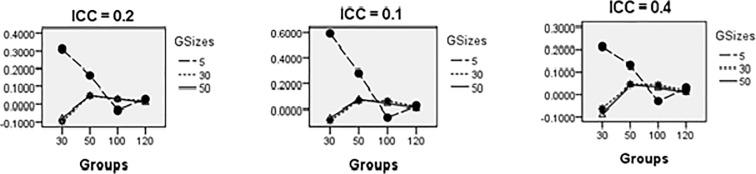
Average relative bias for *σ*_*u*_ across all conditions (ML method).

**Fig 7:**
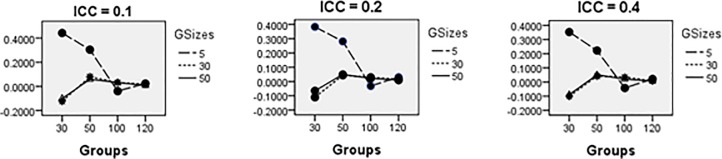
Average relative bias for *σ*_1_ across all conditions (ML method).

Average relative bias of the Multilevel Binary Logistic Model Fixed Effects and Random Effects Estimates across all conditions under ML method of estimation is presented in Figs 1–7. Estimates have negligible bias when the number of groups is large. Figs 1–7 indicate that the bias reduces significantly with the group sizes and the number of groups for all the estimates. Estimates were substantially biased in conditions when the number of groups was 30, group size was 5 and random effects had their smallest values. The relative bias was generally less than 5% when the number of groups was 50. For threshold estimate, the relative bias was negative, and for rest of the fixed effect estimates, the relative bias was positive. Figs 1–7 show that the bias reduces significantly with the number of groups for all the estimates. The group size factor has minimal impact on estimates average relative biases.

Table 1 reflects the influence of the number of groups on multilevel binary logistic model estimates empirical coverage rates under ML method of estimation. This actually indicates a significant effect of the number of groups on the accuracy of estimates standard errors. The largest non-coverage for threshold parameter estimate was 5.8% when the number of groups was 30. Similarly, for *γ*_10_, *γ*_20_, *γ*_01_ and *γ*_11_ the largest non-coverage rates were 5.5%,5.6%,6.1%,6.3% respectively. Furthermore, for *σ*_*u*_ and *σ*_1_ the largest non-coverage rates were 11.2% and 10.6% respectively. The non-coverage rates decreased significantly with increasing the number of groups. The influence of the number of groups was significant on the empirical coverage rate for both fixed effects and random effects estimates.

**Table 1:** 95% CI Coverage rates for the estimates of multilevel binary logistic model by groups (Method = ML).

Parameters	Number of Groups				
	30	50	100	120	P-Value
*γ*_00_ *γ*_10_ *γ*_20_ *γ*_01_ *γ*_11_ *σ*_*u*_ *σ*_1_	0.942 0.945 0.944 0.939 0.937 0.888 0.894	0.943 0.947 0.947 0.939 0.940 0.901 0.910	0.949 0.947 0.951 0.947 0.948 0.908 0.931	0.967 0.964 0.963 0.966 0.961 0.926 0.939	0.0000 0.0000 0.0000 0.0000 0.0000 0.0000 0.0000

Similarly, Table 2 reveals the influence of group size factor on empirical coverage rates in multilevel binary logistic model estimates under ML method of estimation. The group size factor did not play a dominant role in raising the accuracy of standard errors of the estimates. The coverage rates of fixed effects estimates were all acceptable at all group sizes. A separate logistic regression was used to judge the effect of group size levels on estimates empirical coverage rates. P-values indicates the impact of group size factor on both fixed and random effect estimate empirical coverage rates.

**Table 2:** 95% CI Coverage rates for the estimates of multilevel binary logistic model by group size (Method = ML).

Parameters	Group Size			
	5	30	50	P-Value
*γ*_00_ *γ*_10_ *γ*_20_ *γ*_01_ *γ*_11_ *σ*_*u*_ *σ*_1_	0.953 0.955 0.953 0.951 0.951 0.896 0.912	0.951 0.949 0.949 0.946 0.943 0.904 0.922	0.946 0.949 0.952 0.948 0.946 0.916 0.920	0.0116 0.0091 0.7870 0.1910 0.0700 0.0000 0.0335

Moreover, Table 3 shows the influence of ICC on multilevel binary logistic model estimates empirical coverage rates under ML method of estimation. The influence of different levels of ICC was insignificant on empirical coverage rates of both fixed effects and random effects estimates when separate logistic regression was used to judge the effect of different ICC conditions on empirical coverage rates of estimates.

**Table 3:** 95% CI Coverage rates for the estimates of multilevel binary logistic model by ICC (Method = ML).

Parameters	Group Size			
	0.1	0.2	0.4	P-Value
*γ*_00_ *γ*_10_ *γ*_20_ *γ*_01_ *γ*_11_ *σ*_*u*_ *σ*_1_	0.952 0.950 0.949 0.946 0.946 0.901 0.920	0.951 0.951 0.953 0.949 0.948 0.907 0.919	0.948 0.950 0.951 0.948 0.946 0.908 0.918	0.2591 0.9760 0.4710 0.5610 0.9540 0.0519 0.4639

**Fig 8:**
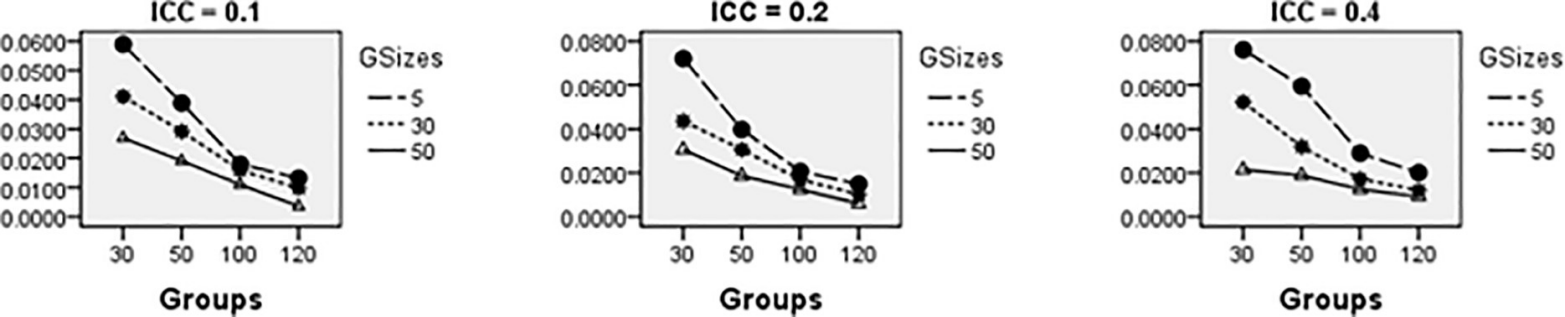
Average relative bias for the estimate of the threshold parameter across all conditions (PQL method).

**Fig 9:**
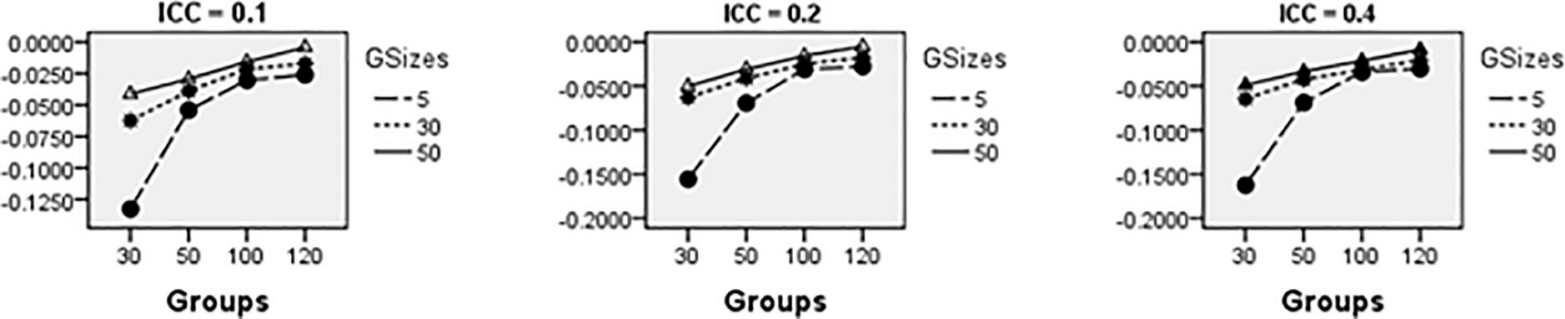
Average relative bias for the estimate of level 1 variable *X*_1*ij*_ coefficient across all conditions (PQL method).

**Fig 10:**
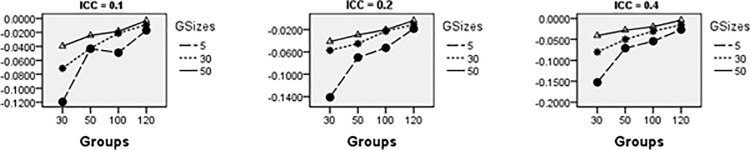
Average relative bias for the estimate of level 1 variable *X*_2*ij*_ coefficient across all conditions (PQL method).

**Fig 11:**
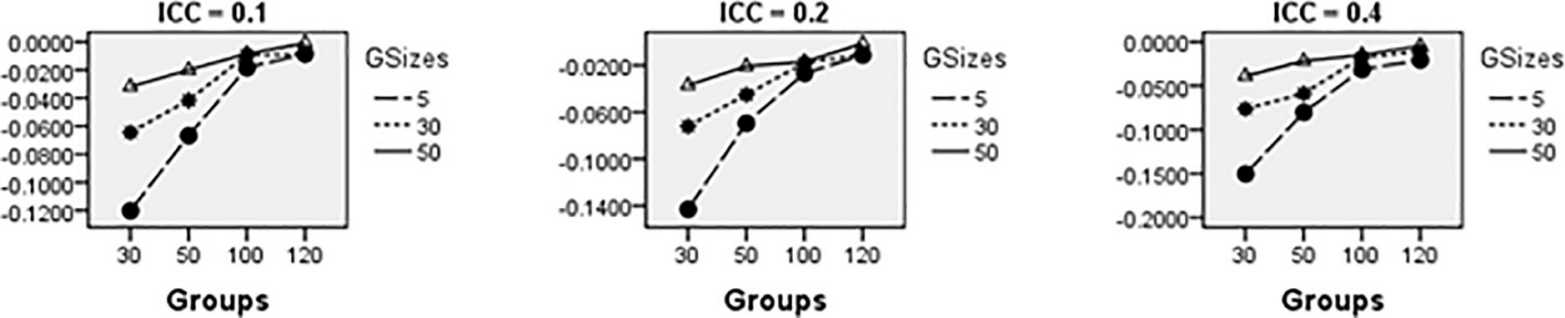
Average relative bias for the estimate of level 2 variable *W*_*j*_ coefficient across all conditions (PQL method).

**Fig 12:**
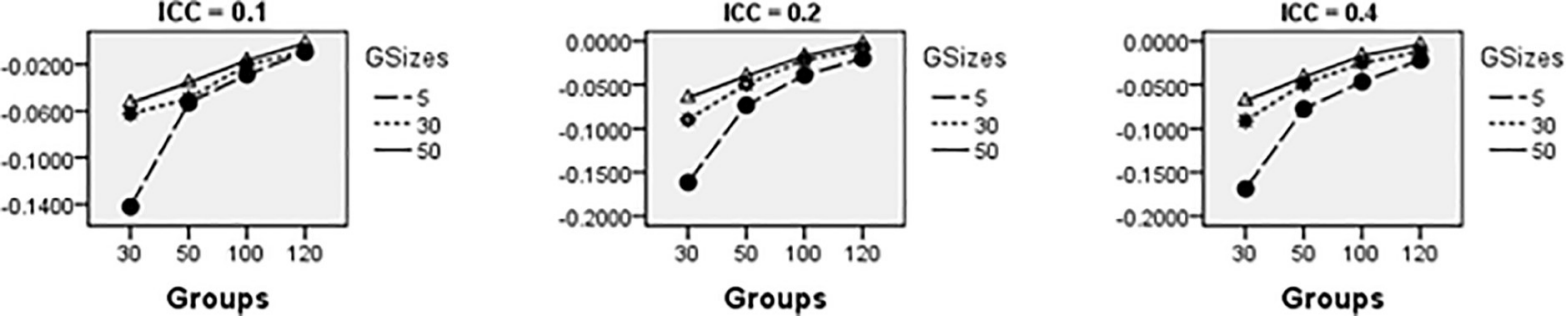
Average relative bias for the estimate of cross-level interaction *X*_1*ij*_*W*_*j*_ coefficient across all conditions (PQL method).

**Fig 13:**
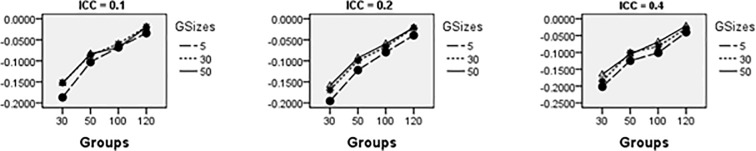
Average relative bias for *σ*_*u*_ across all conditions (PQL method).

**Fig 14:**
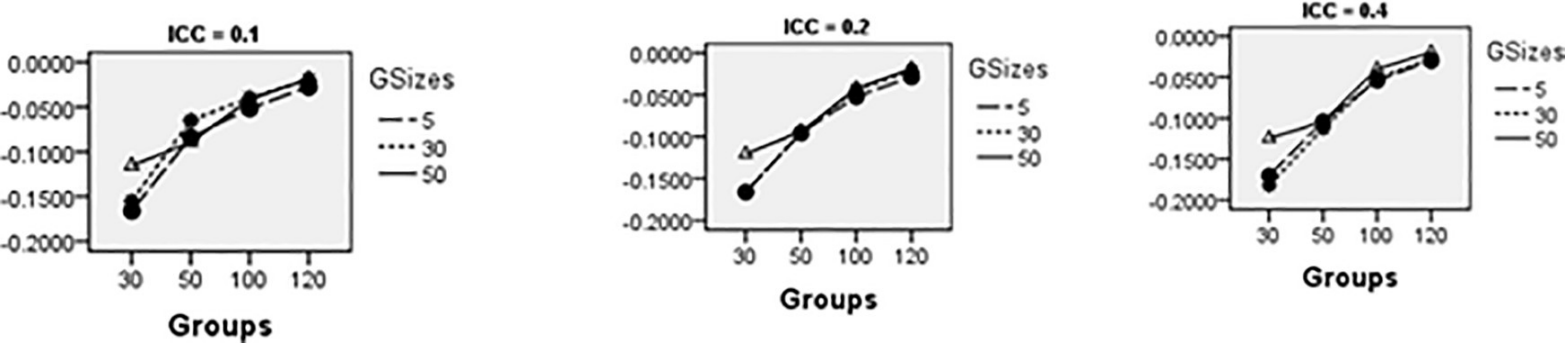
Relative bias for *σ*_1_ across all conditions (PQL method).

Figs 8–14 show the average relative bias for the Multilevel Binary Logistic Model Fixed Effects and Random Effects Estimates across all conditions under PQL method of estimation. It can be observed that the bias reduces significantly with the group sizes and the number of groups for all the estimates.

Table 4 reflects the influence of the number of groups on multilevel binary logistic model fixed effects and random effects estimates empirical coverage rates under PQL method of estimation. The largest non-coverage for the threshold parameter estimate was 8.6% when the number of groups was 30 and it reached 7.2% when the number of groups was 120. Similarly, for *γ*_10_, *γ*_20_, *γ*_01_ and *γ*_11_ the largest non-coverage rates were 8.7%,8.7%,8.8%,9.6% respectively. Furthermore, for *σ*_*u*_ and *σ*_1_ the largest non-coverage rates were 13.5% and 12.6% respectively. The influence of the number of groups was insignificant in most of the conditions when separate logistic regression was used to judge the effect of the number of groups on estimates empirical coverage rates.

**Table 4:** 95% CI Coverage rates for estimates of the multilevel binary logistic model by groups (Method = PQL).

Parameters	Number of Groups				
	30	50	100	120	P-Value
*γ*_00_ *γ*_10_ *γ*_20_ *γ*_01_ *γ*_11_ *σ*_*u*_ *σ*_1_	0.914 0.913 0.913 0.912 0.904 0.865 0.874	0.925 0.916 0.917 0.916 0.909 0.866 0.874	0.927 0.917 0.918 0.917 0.912 0.869 0.875	0.928 0.919 0.921 0.923 0.916 0.872 0.880	0.0007 0.1056 0.0563 0.0132 0.0049 0.1458 0.2361

In the same way, Table 5 reveals the influence of group size factor on multilevel binary logistic model fixed effects and random effects estimates empirical coverage rates under PQL method of estimation. The group size factor played a dominant role in the reduction of estimates non-coverage rates. A separate logistic regression was used to judge the effect of group size factor conditions on estimates empirical coverage rates. The coverage rates were significantly affected by the group size factor. P-values indicate the impact of group size on estimates empirical coverage rates. Additionally, Table 6 highlights the influence of ICC on fixed effects and random effects estimates empirical coverage rates of the multilevel binary logistic model under PQL method of estimation. The influence of different levels of ICC was significant in most of the conditions on both fixed effects and random effects estimates empirical coverage rates when separate logistic regression was used to judge the effect of different levels of ICC on estimates empirical coverage rates.

**Table 5:** 95% CI Coverage rates for estimates of the multilevel binary logistic model by group size (Method = PQL).

Parameters	Group Size			
	5	30	50	P-Value
*γ*_00_ *γ*_10_ *γ*_20_ *γ*_01_ *γ*_11_ *σ*_*u*_ *σ*_1_	0.917 0.905 0.906 0.910 0.900 0.861 0.867	0.924 0.918 0.918 0.918 0.910 0.869 0.876	0.930 0.926 0.928 0.925 0.921 0.874 0.881	0.0000 0.0000 0.0000 0.0000 0.0000 0.0035 0.0000

**Table 6:** 95% CI Coverage rates for estimates of the multilevel binary response variable model by ICC (Method = PQL).

Parameters	Group Size			
	0.1	0.2	0.4	P-Value
*γ*_00_ *γ*_10_ *γ*_20_ *γ*_01_ *γ*_11_ *σ*_*u*_ *σ*_1_	0.929 0.920 0.920 0.919 0.914 0.872 0.879	0.923 0.917 0.917 0.917 0.910 0.868 0.876	0.918 0.912 0.914 0.915 0.906 0.863 0.871	0.0011 0.0223 0.1336 0.1531 0.0493 0.0245 0.0421

Table 7 lists the power rates for multilevel binary logistic model fixed effects estimates under ML method of estimation. The lowest power rates were recorded for all the fixed effects estimates when the number of groups was 30. The power increased substantially with the number of groups. With 100 groups, power rates were well above 0.90.With 120 groups, power rates were 100% in majority of the conditions under ML method. On the contrary, PQL fixed effects estimates power rates were lower than that of ML fixed effects estimates power rates, on average. Power rates increased with the number of groups under both methods of estimation. Table 8 lists the power rates for multilevel binary logistic model fixed effects estimates under PQL method of estimation.

**Table 7:** Power rates for Fixed effects estimates of the multilevel binary response variable model by groups (Method = ML).

Parameters	Number of Groups			
	30	50	100	120
*γ*_00_ *γ*_10_ *γ*_20_ *γ*_01_ *γ*_11_	0.475 0.509 0.491 0.516 0.459	0.756 0.792 0.782 0.802 0.743	0.902 0.911 0.900 0.919 0.914	0.999 1.000 0.999 1.000 1.000

**Table 8:** Power rates for Fixed effects estimates of the multilevel binary response variable model by groups (Method = PQL).

Parameters	Number of Groups			
	30	50	100	120
*γ*_00_ *γ*_10_ *γ*_20_ *γ*_01_ *γ*_11_	0.451 0.465 0.471 0.489 0.442	0.729 0.754 0.749 0.775 0.729	0.876 0.888 0.869 0.891 0.884	0.993 0.996 0.995 0.996 0.995

## Conclusions

In ML method of estimation, the fixed effects estimates were unbiased even with 30 groups; however, the accuracy of the standard errors of fixed effects estimates was achieved when the number of groups was 50. In addition, random effects estimates were underestimated, particularly with 30 groups. Unlike fixed effects estimates standard errors, the accuracy of the random effects estimates standard errors was achieved when the number of groups was 120. Overall, the influence of the number of groups was significant on the accuracy of multilevel binary logistic model estimates and their standard errors. However, group sizes effect was insignificant in most of the conditions on the accuracy of estimates and estimates standard errors. The present study not only confirms (50/50 rule, i.e. minimum of 50 groups and 50 units per group under ML method of estimation) of Moineddin et al.[9] but also suggests that 120 groups and a group size of 50 is mandatory for obtaining of sufficient accuracy of random effects when prevalence of the outcome is around 20 percent. Additionally, the influence of the number of groups was substantial on empirical power rates of fixed effects estimates under ML method of estimation. The power rates for all the fixed effects estimates increased with an increase in the number of groups. The results obtained in this study are parallel to the previous studies when the response variable is continuous [22–23].

On the other hand, the fixed effects estimates had the largest biases when group size was at the lowest, i.e. 5 under PQL method of estimation. However, their biases were negligible when group size was 50. Unlike the ML method of estimation, the group size was the most significant factor that influenced the accuracy of multilevel binary logistic model estimates and estimates standard errors. Furthermore, the accuracy of the fixed effects estimates standard errors was not satisfactory even with a group size of 50. Similarly, the random effects estimates and their standard errors were under estimated under PQL method of estimation. Random effects estimates standard errors accuracy was far behind than that of ML method across all conditions. The impact of the number of groups in most of the conditions was insignificant on the accuracy of estimates standard errors. Therefore, a (50/60 rule, i.e. minimum of 50 groups and 60 units per group under PQL method of estimation) is recommended to achieve sufficient accuracy. In addition, it is also recommended that 120 groups and a group size of 70 may be used to achieve sufficient accuracy for the variance components estimates and their standard errors when prevalence of the outcome is around 20 percent. Larger ICC values also decreased the accuracy of estimates and their standard errors. Similarly, like ML method of estimation, the power rates for the multilevel binary logistic regression model fixed parameter estimates increased with the number of groups. The power rates of PQL method of estimation was also on the lower side as compared to ML method power rates.

Across all conditions, PQL method estimates and estimates standard errors of multilevel binary logistic model are not comparable to that of ML method. On the basis of the present study results, it is, therefore, recommended that PQL method for binary outcome variable may be avoided in situations such as low prevalence of outcome, larger values of random effects and even when group sizes are 50 or less. However, the significance of Penalized Quasi Likelihood method of estimation was ignored earlier which proved to be an extremely effective method when random effects are small.
