# Supplementary material for: Sample size issues in multilevel logistic regression models
Source: PLoS One. 2019 Nov 22;14(11):e0225427. doi: 10.1371/journal.pone.0225427 (PMC6874355; doi:10.1371/journal.pone.0225427)
Supplement: Attachment — Submitted filename: Response to Reviewers.docx [file pone.0225427.s001.docx]

**Rebuttal letter for revision of paper to PLOS ONE journal**

Dr. Sajjad Ahmad Khan
Associate Professor
Department of Statistics,

Islamia College Peshawar, Pakistan

November 24, 2019

Dear Prof. Dr. Feng Chen,

We are thankful to you and the reviewers for valuable comments and suggestions on our manuscript title “**SAMPLE SIZE ISSUES IN MULTILEVEL LOGISTIC REGRESSION MODELS**”. We have edited the manuscript to address the comments/suggestions.

**Response to the comments of reviewer 1 is as under:**

Comments of Reviewer #1

This study focuses on dealing with the sample size issue in multilevel Logistic regression models, which is important for the applications of these models in practice. While it is worth of investigation, the authors should highlight their academic contributions more significantly. At least, the gap between the current research and the previous should be stated clearly in the Section of Introduction. Some other more detailed comments are as follows:

1. The authors should number lines in the manuscript for the convenience of paper review.

2. The authors investigated the same size issue for maximum likelihood and penalized quasi-likelihood estimation methods separately. While these estimation methods are popular, Bayesian hierarchical modeling has also gained much prevalence in recent years. Under Bayesian hierarchical modeling framework, not only the multilevel structure but also spatial and temporal correlations are accounted for, which can reduce model misspecification and estimation bias significantly. The authors should review some representative theoretical works and their practical applications. For example, the following papers on Bayesian hierarchical/spatial-temporal modeling can be acknowledged in the section of Introduction:

Zeng Q., Gu W., Zhang X., Wen H., Lee J., Hao W. (2019). Analyzing freeway crash severity using a Bayesian spatial generalized ordered logit model with conditional autoregressive priors. Accident Analysis & Prevention, 127, 87-95.
Zeng Q., Wen H., Huang H., Pei X., Wong S.C. (2018). Incorporating temporal correlation into a multivariate random parameters Tobit model for modeling crash rate by injury severity. Transportmetrica A: Transport Science, 14 (3): 177-191.
Zeng Q., Guo Q., Wong S.C., Wen H., Huang H., Pei X., (2019). Incorporating temporal correlation into a multivariate random parameters Tobit model for modeling crash rate by injury severity. Transportmetrica A: Transport Science, 15 (2): 1867-1884.

3. In the Result Section, the authors are suggested to illustrate the findings with references to those in the previous, to further justify the reasonableness of the results. Especially, the differences between the results for the two estimation methods should be explained explicitly, as it may be a significant potential contribution of this research.

4. The figures shown in the manuscript are not discussed in the text.

5. Language editing is required, because there are many grammar errors and improper expressions in the manuscript.

Response to reviewer #1

So far, very little research has been conducted in the literature regarding sample size determination in the context of multilevel logistic regression models. For example, there is nothing in the contemporary research about PQL method in multilevel logistic regression models. Therefore, the present study attempts to capitalize on a novel state-of-the-art rule that encompasses all the weaknesses of the available methods for both fixed and random effects estimates under ML and PQL methods of estimation. In addition, the present study also provides guidelines about optimum sample size needed for multilevel logistic regression models. Random intercept and random slope model with two level-1 and one level-2 explanatory variables using threshold parameter concept are used. Further, larger random effects are incorporated in the present study, which were unnoticed in the previous literature. Moreover, relevant factor and their levels were also ignored in [9] and [10]. A detailed comparison of ML method and PQL method has been made in terms of sample size. Response to some other detailed comments are as under:

1. The lines have been numbered in the revised manuscript.
2. The papers on Bayesian hierarchical/spatial-temporal modeling suggested by the reviewer # 1 have been reviewed and acknowledged in the section of Introduction.
3. The difference between the results for the two estimation methods has been discussed.
4. Discussion on figures has been incorporated.
5. Language editing has been done and errors have been removed.

**Response to the comments of reviewer 2 is as under:**

Comments of Reviewer #2

The manuscript attempts to study the requirements on sample sizes in multilevel logistic regression models. The manuscript is overall well written and structured. There are, however, some revisions required before it can be considered for publication.
1. The authors state that the objective of the study is to determine the optimal sample size of multilevel logistic regression. Nevertheless, the abstract seems to be gear towards the comparison between ML and PQL estimations. The abstract should discuss more about the findings on sample sizes in addition to the difference between ML and PQL methods.

2. Since Plos One has a readership with various background instead of statistics and econometrics. The authors should explain the acronyms at its first mention. For example, In page 2, Line9, ICC should be explained at its first mention.

3. In explaining the wide use of multilevel models, the authors are suggested to cite references that adopts multilevel models in other areas. The following literature should be discussed and acknowledged in the literature:
[1] Feng Chen, Haorong Peng, Xiaoxiang Ma, Jieyu Liang, Wei Hao, Xiaodong Pan(2019) “Examining the safety of trucks under crosswind at bridge-tunnel section: A driving simulator study”, Tunnelling and Underground Space Technology, 92, 103034. [https://doi.org/10.1016/j.tust.2019.103034](https://doi.org/10.1016/j.tust.2019.103034)
[2] F. Chen and S. R. Chen (2011). “Injury severities of truck drivers in single- and multi-vehicle accidents on rural highway”, Accident Analysis and Prevention, 43（5）, 1677-1688.
[3] Feng Chen, Mingtao Song and Xiaoxiang Ma (2019), Investigation on the Injury Severity of Drivers in Rear-End Collisions Between Cars Using a Random Parameters Bivariate Ordered Probit Model, International Journal of Environmental Research and Public Health, 16(14) , 2632.

4. I have some concerns about the simulation design. The authors only considered samples with large group number but small group sizes. Why don’t the authors consider small group number with large sizes? The latter is usually encountered in some areas.

5. The authors didn’t consider Bayesian methods, as pointed out by Gelman and Hill, even samples with small group number and small group sizes can benefit from multilevel model under Bayesian framework.
Reference: Gelman, Andrew, and Jennifer Hill. Data analysis using regression and multilevel/hierarchical models. Cambridge university press, 2006.

Response to reviewer #2

1. Main findings of the study have been discussed in the abstract.
2. ICC has been defined.
3. The papers suggested by the reviewer # 2 have been discussed and acknowledged in the manuscript.
4. The accuracy of the standard errors of variance estimates in multilevel logistic regression models needs large number of groups [9, 10]. That is why relatively large group number and moderate group sizes have been considered in the present study.
5. Bayesian methods may also be very useful in such situations and can reduce model misspecification and estimation bias significantly. However, these can be considered in the future studies.

Thank you for your consideration of this manuscript.

Sincerely,

Dr. Sajjad Ahmad Khan
