# Supplementary material for: Sample size issues in multilevel logistic regression models
Source: PLoS One. 2019 Nov 22;14(11):e0225427. doi: 10.1371/journal.pone.0225427 (PMC6874355; doi:10.1371/journal.pone.0225427)
Supplement: Peer review history

# Peer review: Decision Letter 0

**DOI:** 10.1371/journal.pone.0225427.r001

**Authors:** Feng Chen

11 Sep 2019

PONE-D-19-14953

SAMPLE SIZE ISSUES IN MULTILEVEL LOGISTIC REGRESSION MODELS

PLOS ONE

Dear Dr. Khan,

Thank you for submitting your manuscript to PLOS ONE. After careful consideration, we feel that it has merit but does not fully meet PLOS ONE’s publication criteria as it currently stands. Therefore, we invite you to submit a revised version of the manuscript that addresses the points raised during the review process.

We would appreciate receiving your revised manuscript by Oct 26 2019 11:59PM. When you are ready to submit your revision, log on to https://www.editorialmanager.com/pone/ and select the 'Submissions Needing Revision' folder to locate your manuscript file.

If you would like to make changes to your financial disclosure, please include your updated statement in your cover letter.

To enhance the reproducibility of your results, we recommend that if applicable you deposit your laboratory protocols in protocols.io, where a protocol can be assigned its own identifier (DOI) such that it can be cited independently in the future. For instructions see: http://journals.plos.org/plosone/s/submission-guidelines#loc-laboratory-protocols

Please include the following items when submitting your revised manuscript:

- A rebuttal letter that responds to each point raised by the academic editor and reviewer(s). This letter should be uploaded as separate file and labeled 'Response to Reviewers'.
- A marked-up copy of your manuscript that highlights changes made to the original version. This file should be uploaded as separate file and labeled 'Revised Manuscript with Track Changes'.
- An unmarked version of your revised paper without tracked changes. This file should be uploaded as separate file and labeled 'Manuscript'.

Please note while forming your response, if your article is accepted, you may have the opportunity to make the peer review history publicly available. The record will include editor decision letters (with reviews) and your responses to reviewer comments. If eligible, we will contact you to opt in or out.

We look forward to receiving your revised manuscript.

Kind regards,

Feng Chen

Academic Editor

PLOS ONE

Journal Requirements:

1.  When submitting your revision, we need you to address these additional requirements.

Please ensure that your manuscript meets PLOS ONE's style requirements, including those for file naming. The PLOS ONE style templates can be found at

http://www.journals.plos.org/plosone/s/file?id=wjVg/PLOSOne_formatting_sample_main_body.pdf and http://www.journals.plos.org/plosone/s/file?id=ba62/PLOSOne_formatting_sample_title_authors_affiliations.pdf

[Note: HTML markup is below. Please do not edit.]

Reviewers' comments:

Reviewer's Responses to Questions

**Comments to the Author**

1. Is the manuscript technically sound, and do the data support the conclusions?

The manuscript must describe a technically sound piece of scientific research with data that supports the conclusions. Experiments must have been conducted rigorously, with appropriate controls, replication, and sample sizes. The conclusions must be drawn appropriately based on the data presented. 

Reviewer #1: Partly

Reviewer #2: Partly

**********

2. Has the statistical analysis been performed appropriately and rigorously? 

Reviewer #1: Yes

Reviewer #2: Yes

**********

3. Have the authors made all data underlying the findings in their manuscript fully available?

The PLOS Data policy requires authors to make all data underlying the findings described in their manuscript fully available without restriction, with rare exception (please refer to the Data Availability Statement in the manuscript PDF file). The data should be provided as part of the manuscript or its supporting information, or deposited to a public repository. For example, in addition to summary statistics, the data points behind means, medians and variance measures should be available. If there are restrictions on publicly sharing data—e.g. participant privacy or use of data from a third party—those must be specified.

Reviewer #1: Yes

Reviewer #2: Yes

**********

4. Is the manuscript presented in an intelligible fashion and written in standard English?

PLOS ONE does not copyedit accepted manuscripts, so the language in submitted articles must be clear, correct, and unambiguous. Any typographical or grammatical errors should be corrected at revision, so please note any specific errors here.

Reviewer #1: No

Reviewer #2: Yes

**********

5. Review Comments to the Author

Please use the space provided to explain your answers to the questions above. You may also include additional comments for the author, including concerns about dual publication, research ethics, or publication ethics. (Please upload your review as an attachment if it exceeds 20,000 characters)

Reviewer #1: This study focuses on dealing with the sample size issue in multilevel Logistic regression models, which is important for the applications of these models in practice. While it is worth of investigation, the authors should highlight their academic contributions more significantly. At least, the gap between the current research and the previous should be stated clearly in the Section of Introduction. Some other more detailed comments are as follows:

1. The authors should number lines in the manuscript for the convenience of paper review.

2. The authors investigated the same size issue for maximum likelihood and penalized quasi-likelihood estimation methods separately. While these estimation methods are popular, Bayesian hierarchical modeling has also gained much prevalence in recent years. Under Bayesian hierarchical modeling framework, not only the multilevel structure but also spatial and temporal correlations are accounted for, which can reduce model misspecification and estimation bias significantly. The authors should review some representative theoretical works and their practical applications. For example, the following papers on Bayesian hierarchical/spatial-temporal modeling can be acknowledged in the section of Introduction:

Zeng Q., Gu W., Zhang X., Wen H., Lee J., Hao W. (2019). Analyzing freeway crash severity using a Bayesian spatial generalized ordered logit model with conditional autoregressive priors. Accident Analysis & Prevention, 127, 87-95.

Zeng Q., Wen H., Huang H., Pei X., Wong S.C. (2018). Incorporating temporal correlation into a multivariate random parameters Tobit model for modeling crash rate by injury severity. Transportmetrica A: Transport Science, 14 (3): 177-191.

Zeng Q., Guo Q., Wong S.C., Wen H., Huang H., Pei X., (2019). Incorporating temporal correlation into a multivariate random parameters Tobit model for modeling crash rate by injury severity. Transportmetrica A: Transport Science, 15 (2): 1867-1884.

3. In the Result Section, the authors are suggested to illustrate the findings with references to those in the previous, to further justify the reasonableness of the results. Especially, the differences between the results for the two estimation methods should be explained explicitly, as it may be a significant potential contribution of this research.

4. The figures shown in the manuscript are not discussed in the text.

5. Language editing is required, because there are many grammar errors and improper expressions in the manuscript.

Reviewer #2: The manuscript attempts to study the requirements on sample sizes in multilevel logistic regression models. The manuscript is overall well written and structured. There are, however, some revisions required before it can be considered for publication.

1. The authors state that the objective of the study is to determine the optimal sample size of multilevel logistic regression. Nevertheless, the abstract seems to be gear towards the comparison between ML and PQL estimations. The abstract should discuss more about the findings on sample sizes in addition to the difference between ML and PQL methods.

2. Since Plos One has a readership with various background instead of statistics and econometrics. The authors should explain the acronyms at its first mention. For example, In page 2, Line9, ICC should be explained at its first mention.

3. In explaining the wide use of multilevel models, the authors are suggested to cite references that adopts multilevel models in other areas. The following literature should be discussed and acknowledged in the literature:

[1] Feng Chen, Haorong Peng, Xiaoxiang Ma, Jieyu Liang, Wei Hao, Xiaodong Pan(2019) “Examining the safety of trucks under crosswind at bridge-tunnel section: A driving simulator study”, Tunnelling and Underground Space Technology, 92, 103034. https://doi.org/10.1016/j.tust.2019.103034

[2] F. Chen and S. R. Chen (2011). “Injury severities of truck drivers in single- and multi-vehicle accidents on rural highway”, Accident Analysis and Prevention, 43（5）, 1677-1688.

[3] Feng Chen, Mingtao Song and Xiaoxiang Ma (2019), Investigation on the Injury Severity of Drivers in Rear-End Collisions Between Cars Using a Random Parameters Bivariate Ordered Probit Model, International Journal of Environmental Research and Public Health, 16(14) , 2632.

4. I have some concerns about the simulation design. The authors only considered samples with large group number but small group sizes. Why don’t the authors consider small group number with large sizes? The latter is usually encountered in some areas.

5. The authors didn’t consider Bayesian methods, as pointed out by Gelman and Hill, even samples with small group number and small group sizes can benefit from multilevel model under Bayesian framework.

Reference: Gelman, Andrew, and Jennifer Hill. Data analysis using regression and multilevel/hierarchical models. Cambridge university press, 2006.

**********

6. PLOS authors have the option to publish the peer review history of their article (what does this mean?). If published, this will include your full peer review and any attached files.

If you choose “no”, your identity will remain anonymous but your review may still be made public.

**Do you want your identity to be public for this peer review?** For information about this choice, including consent withdrawal, please see our Privacy Policy.

Reviewer #1: No

Reviewer #2: No

[NOTE: If reviewer comments were submitted as an attachment file, they will be attached to this email and accessible via the submission site. Please log into your account, locate the manuscript record, and check for the action link "View Attachments". If this link does not appear, there are no attachment files to be viewed.]

While revising your submission, please upload your figure files to the Preflight Analysis and Conversion Engine (PACE) digital diagnostic tool, https://pacev2.apexcovantage.com/. PACE helps ensure that figures meet PLOS requirements. To use PACE, you must first register as a user. Registration is free. Then, login and navigate to the UPLOAD tab, where you will find detailed instructions on how to use the tool. If you encounter any issues or have any questions when using PACE, please email us at figures@plos.org. Please note that Supporting Information files do not need this step.

# Author comment: Author response to Decision Letter 0

**DOI:** 10.1371/journal.pone.0225427.r002

28 Oct 2019

The following three files have been uploaded.

1. Revised manuscript with track changes

2. Manuscript

3. Response to reviewers

Attachment Submitted filename: Response to Reviewers.docx Click here for additional data file.

# Peer review: Decision Letter 1

**DOI:** 10.1371/journal.pone.0225427.r003

**Authors:** Feng Chen

6 Nov 2019

SAMPLE SIZE ISSUES IN MULTILEVEL LOGISTIC REGRESSION MODELS

PONE-D-19-14953R1

Dear Dr. Khan,

We are pleased to inform you that your manuscript has been judged scientifically suitable for publication and will be formally accepted for publication once it complies with all outstanding technical requirements.

Within one week, you will receive an e-mail containing information on the amendments required prior to publication. When all required modifications have been addressed, you will receive a formal acceptance letter and your manuscript will proceed to our production department and be scheduled for publication.

Shortly after the formal acceptance letter is sent, an invoice for payment will follow. To ensure an efficient production and billing process, please log into Editorial Manager at https://www.editorialmanager.com/pone/, click the "Update My Information" link at the top of the page, and update your user information. If you have any billing related questions, please contact our Author Billing department directly at authorbilling@plos.org.

If your institution or institutions have a press office, please notify them about your upcoming paper to enable them to help maximize its impact. If they will be preparing press materials for this manuscript, you must inform our press team as soon as possible and no later than 48 hours after receiving the formal acceptance. Your manuscript will remain under strict press embargo until 2 pm Eastern Time on the date of publication. For more information, please contact onepress@plos.org.

With kind regards,

Feng Chen

Academic Editor

PLOS ONE

Additional Editor Comments (optional):

Reviewers' comments:

Reviewer's Responses to Questions

**Comments to the Author**

1. If the authors have adequately addressed your comments raised in a previous round of review and you feel that this manuscript is now acceptable for publication, you may indicate that here to bypass the “Comments to the Author” section, enter your conflict of interest statement in the “Confidential to Editor” section, and submit your "Accept" recommendation.

Reviewer #1: (No Response)

Reviewer #2: All comments have been addressed

**********

2. Is the manuscript technically sound, and do the data support the conclusions?

The manuscript must describe a technically sound piece of scientific research with data that supports the conclusions. Experiments must have been conducted rigorously, with appropriate controls, replication, and sample sizes. The conclusions must be drawn appropriately based on the data presented. 

Reviewer #1: (No Response)

Reviewer #2: Yes

**********

3. Has the statistical analysis been performed appropriately and rigorously? 

Reviewer #1: (No Response)

Reviewer #2: Yes

**********

4. Have the authors made all data underlying the findings in their manuscript fully available?

The PLOS Data policy requires authors to make all data underlying the findings described in their manuscript fully available without restriction, with rare exception (please refer to the Data Availability Statement in the manuscript PDF file). The data should be provided as part of the manuscript or its supporting information, or deposited to a public repository. For example, in addition to summary statistics, the data points behind means, medians and variance measures should be available. If there are restrictions on publicly sharing data—e.g. participant privacy or use of data from a third party—those must be specified.

Reviewer #1: (No Response)

Reviewer #2: Yes

**********

5. Is the manuscript presented in an intelligible fashion and written in standard English?

PLOS ONE does not copyedit accepted manuscripts, so the language in submitted articles must be clear, correct, and unambiguous. Any typographical or grammatical errors should be corrected at revision, so please note any specific errors here.

Reviewer #1: (No Response)

Reviewer #2: Yes

**********

6. Review Comments to the Author

Please use the space provided to explain your answers to the questions above. You may also include additional comments for the author, including concerns about dual publication, research ethics, or publication ethics. (Please upload your review as an attachment if it exceeds 20,000 characters)

Reviewer #1: The authors should be thanked for the efforts on improving the manuscript. All my comments have been addressed properly.

Reviewer #2: (No Response)

**********

7. PLOS authors have the option to publish the peer review history of their article (what does this mean?). If published, this will include your full peer review and any attached files.

If you choose “no”, your identity will remain anonymous but your review may still be made public.

**Do you want your identity to be public for this peer review?** For information about this choice, including consent withdrawal, please see our Privacy Policy.

Reviewer #1: No

Reviewer #2: No

# Editor report: Acceptance letter

**DOI:** 10.1371/journal.pone.0225427.r004

**Authors:** Feng Chen

13 Nov 2019

PONE-D-19-14953R1 

SAMPLE SIZE ISSUES IN MULTILEVEL LOGISTIC REGRESSION MODELS 

Dear Dr. Khan:

I am pleased to inform you that your manuscript has been deemed suitable for publication in PLOS ONE. Congratulations! Your manuscript is now with our production department. 

If your institution or institutions have a press office, please notify them about your upcoming paper at this point, to enable them to help maximize its impact. If they will be preparing press materials for this manuscript, please inform our press team within the next 48 hours. Your manuscript will remain under strict press embargo until 2 pm Eastern Time on the date of publication. For more information please contact onepress@plos.org.

For any other questions or concerns, please email plosone@plos.org. 

Thank you for submitting your work to PLOS ONE.

With kind regards,

PLOS ONE Editorial Office Staff

on behalf of

Dr. Feng Chen 

Academic Editor

PLOS ONE
